# The influence of an intermediate care hospital on health care utilization among elderly patients - a retrospective comparative cohort study

**DOI:** 10.1186/s12913-015-0708-4

**Published:** 2015-02-01

**Authors:** Unni Dahl, Roar Johnsen, Rune Sætre, Aslak Steinsbekk

**Affiliations:** Department of Public Health and General Practice, Norwegian University of Science and Technology, Medisinsk teknisk forskningssenter, Post box 8905 , 7491 Trondheim, Norway; Central Norway Health Authority, 7500 Stjørdal, Norway

**Keywords:** Intermediate care hospital, Hospital discharge, Health care utilization, Length of stay, Readmission, Primary health care, Elderly

## Abstract

**Background:**

An intermediate care hospital (ICH) was established in a municipality in Central Norway in 2007 to improve the coordination of services and follow-up among elderly and chronically ill patients after hospital discharge. The aim of this study was to compare health care utilization by elderly patients in a municipality with an ICH to that of elderly patients in a municipality without an ICH.

**Methods:**

This study was a retrospective comparative cohort study of all hospitalized patients aged 60 years or older in two municipalities. The data were collected from the national register of hospital use from 2005 to 2012, and from the local general hospital and two primary health care service providers from 2008 to 2012 (approx. 1,250 patients per follow-up year). The data were analyzed using descriptive statistics and analysis of covariance (ANCOVA).

**Results:**

The length of hospital stay decreased from the time the ICH was introduced and remained between 10% and 22% lower than the length of hospital stay in the comparative municipality for the next five years. No differences in the number of readmissions or admissions during one year follow-up after the index stay at the local general hospital or changes in primary health care utilization were observed. In the year after hospital discharge, the municipality with an ICH offered more hour-based care to elderly patients living at home (estimated mean = 234 [95% CI 215-252] versus 175 [95% CI 154-196] hours per person and year), while the comparative municipality had a higher utilization of long-term stays in nursing homes (estimated mean = 33.3 [95% CI 29.0-37.7] versus 21.9 [95% CI 18.0-25.7] days per person and year).

**Conclusions:**

This study indicates that the introduction of an ICH rapidly reduces the length of hospital stay without exposing patients to an increased health risk. The ICH appears to operate as an extension of the general hospital, with only a minor impact on the pattern of primary health care utilization.

## Background

Providing optimal health care services for an aging population is a major concern in many countries [1-3]. In particular, elderly patients with reduced functionality who are ready for discharge represent a challenge with respect to providing relevant follow-up [4-6]. These patients may benefit from discharge planning and post-discharge support [7,8].

Previous research has indicated that poor discharge planning and inadequate coordination of care may result in increased readmissions [9,10]. Hence, as the length of hospital stays become shorter, questions about patient safety emerge [11]. The pressure to achieve rapid hospital throughput has raised concerns that elderly patients are being prematurely discharged and will be subsequently readmitted [12]. Thus, various approaches and interventions concerning discharge preparation and follow-up have been tested [13-15].

Intermediate care is a new healthcare model between hospital and home that originated in the UK [16]; and similar models have been developed in other countries [17-20]. Intermediate services are targeted primarily at preventing unnecessary hospital use and maximizing independent living for elderly people [21]. The arrangements are time-limited and range from nurse-led units, nursing home-based rehabilitation and community hospitals to care delivered in the patients’ own homes. Some evidence is available concerning the effect of such arrangements [22]. A Cochrane review found that patients allocated to intermediate care in nursing-led units tended to be better prepared for discharge and had a lower rate of readmission soon after discharge [23]. Intermediate care in community hospitals has also been found to decrease the number of readmissions and to facilitate greater independence for elderly people [17,24,25]. Another review demonstrated that the rate of readmission increased for elderly patients with a mix of conditions when they were allocated to “hospital at home” rather than to in-patient hospital care [26].

Although no clear evidence is available to support the fear that intermediate care leads to inadequate rehabilitation for older people [26,27], evaluations of intermediate care are not conclusive [28,29]. Future studies of the effects of specific intermediate services and implementation research are recommended [23,26].

There is a lack of studies that investigate the development of health care utilization over time in municipalities that integrate a specific intermediate care service as a part of primary health care. Therefore, the aim of this study was to investigate whether a municipality with an intermediate care hospital (ICH) that provides support after hospital discharge has different health care utilization rates and a different risk of readmission for persons aged 60 or older than a municipality without an ICH. This topic was investigated in three research aims:

1. Identify changes in total hospital use before and after the establishment of an intermediate care hospital (2005 to 2012).
2. Compare readmissions to a local general hospital during a 4-year period (starting at index stay 2008–2011 with follow-up ending in 2012).
3. Compare the use of a local general hospital and the use of primary health care services one year after hospitalization during a 4-year period (starting at index stay 2008–2011 with follow-up ending in 2012).

## Methods

### Design

This study was a register-based retrospective cohort study using data from national registers for the period from 2005 to 2012 and from different health care providers for the period from 2008 to 2012.

The Regional Committee for Medical Research Ethics approved the study (2009/1697a). The committee gave permission to use the data, which was de-identified, without obtaining individual consent. This required that data on the use of primary health care was not linked to the use of hospital services on an individual level. Accordingly, the data of the cohort and the subgroup are reported separately.

### Setting

The health and social care system in Norway is primarily a public system. Specialist services, which are predominately provided by hospitals, are state-owned and organized within Health Enterprises [30]. The municipalities are responsible for primary health care services and have considerable freedom in the organization of the services, resulting in varying health care strategies and priorities [31,32]. For example, large differences in the proportion of elderly patients living in nursing homes and elderly receiving assistance at home exist between some municipalities.

**Figure 1:**
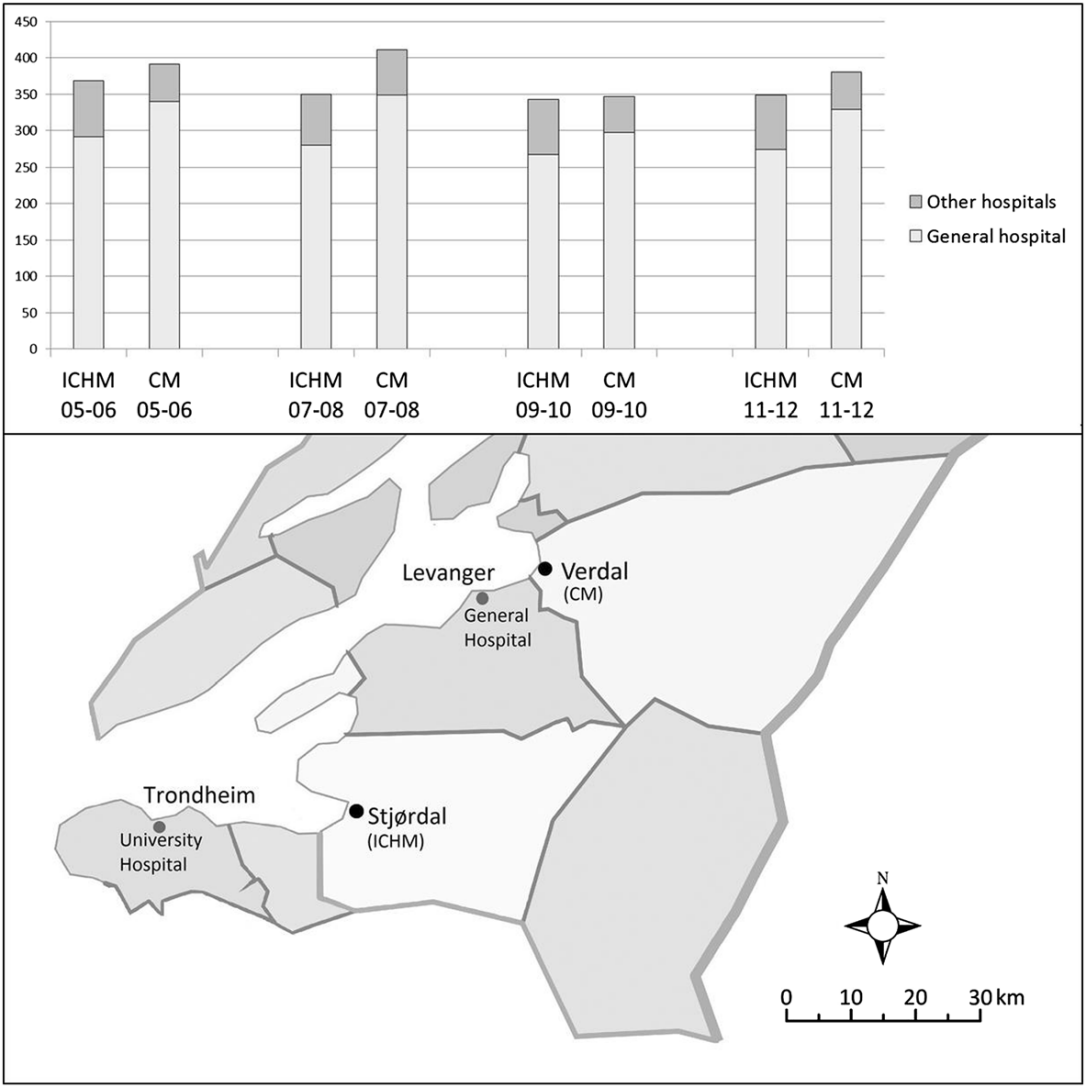
**Total hospital admission.** The histogram presents the average two-year age-standardized hospital admission rates to the local general hospital and to other Norwegian hospitals per 1,000 inhabitants aged 60+ residing in the ICHM (Stjørdal) and the CM (Verdal) in 2005–2012. Source: NPR. The map illustrates the distances between the municipalities, the local general hospital and the university hospital (the two municipalities’ main urban centers are marked). ICHM (Intermediate Care Hospital Municipality). CM (Comparative Municipality).

The cohort used in this study originates from two municipalities in Central Norway. The Intermediate Care Hospital Municipality (ICHM) and the Comparative Municipality (CM) are located in the catchment area of a 200-bed general hospital named “Sykehuset Levanger” within the Health Enterprise “Helse Nord-Trøndelag”. The hospital has medical, surgical, rehabilitation and gynecological departments. The distances between the local general hospital and the ICHM’s and CM’s urban centers are 46 km and 16 km, respectively (Figure 1). Between 2005 and 2012, the local general hospital captured 77-80% of all the hospitalizations for persons aged 60 years or older (60+) who resided in the ICHM, compared to 83-87% for this population in the CM (Figure 1). In addition, the admission rates to the university hospital in the region were 16%-20% for persons aged 60+ who resided in the ICHM from 2008 to 2012 and 10%-14% for this population in the CM (unpublished data, collected from the regional university hospital’s register). Only minor differences in the hospital admission rates to the local general hospital and to other hospitals were observed from 2005 through 2012 (Figure 1). During this period, there was a small reduction in the admission rates in both municipalities, but the CM rate was consistently higher than the ICHM rate.

In 2012, the population size was 22,100 in the ICHM and 14,400 in the CM. In the ICHM 21% of the inhabitants were aged 60+ compared to 23% in the CM. The municipalities had a comparable age and gender distribution for persons aged 60+ from 2005 through 2012. The mean age of women and men aged 60+ in both municipalities was 72–73 years and 70–71 years, respectively.

The ICHM established an intermediate care hospital (ICH) in collaboration with the general hospital and the regional health authorities on March 1, 2007 [33]. The ICH is a 12-bed unit co-located with the primary health care service; this unit was fully operational in 2008. The objective of the unit is to improve the coordination of services and follow-up after hospital discharge among elderly and chronically ill patients [34]. The average length of stay in the ICH was 11 days in 2012 (unpublished data). After the ICH stay, the patients are discharged to their homes, with or without assistance from home care services, or to a nursing home.

Patients from the CM receive “care as usual”. They are hospitalized until discharge to their homes, with or without assistance from home care services, or to a nursing home.

The proportion of available institutional care beds for inhabitants in the ICHM aged 60+ was 1.5% (including the ICH), while it was 3.4% in the CM (2012).

### Samples and data collection

**Figure 2:**
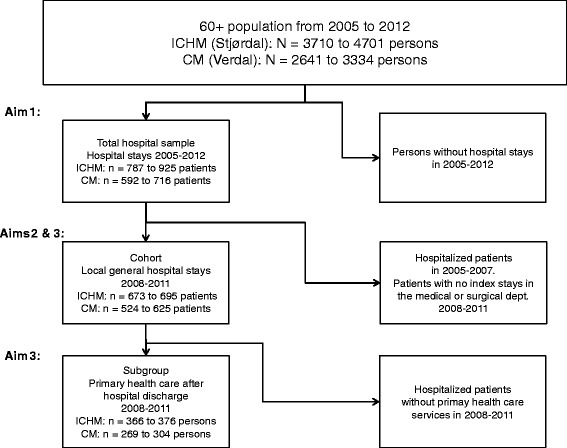
**Three samples: The total hospital sample, the cohort and the subgroup.** The total hospital sample, the cohort and the subgroup were extracted from the 60+ populations of the ICHM and the CM between 2005 and 2012. ICHM (Intermediate Care Hospital Municipality). CM (Comparative Municipality).

As shown in Figure 2, three samples were extracted from the population of the ICHM and the CM to answer the different research aims.

Sample 1: The total hospital sample was used to address aim 1. This sample identified hospital use before and after the establishment of the ICH and consisted of in-patient admissions to any national hospital (2005–2012) for persons aged 60+ who resided in the ICHM or the CM. The data was obtained from the Norwegian Patient Register (NPR).

Sample 2: The cohort of patients aged 60+ at the local general hospital was used to address aims 2 and 3 (to compare readmissions and the utilization of the local general hospital between the two municipalities). This cohort included in-patients admitted to the medical or surgical department from January 1, 2008 to December 31, 2011.These two departments accounted for 96-98% of all in-patient admissions. During the patients’ follow-up year, all subsequent in-patient admissions to this hospital were also registered (this included the rehabilitation and gynecological departments). The data was collected from the general hospital’s local register for the years 2008 through 2012.

Sample 3: A subgroup was used to address aim 3 (to compare the utilization of primary health care services between the two municipalities). This subgroup consisted of persons aged 60+ who received primary health care services within one year after discharge from the local general hospital (medical or surgical department) from January 1, 2008 to December 31, 2011. Aggregated data for all primary health care users aged 60+ and for this subgroup were collected from the registers of the municipalities from 2008 through 2012. Data related to the ICH stays were collected from the ICH and subsequently added to the primary health care services for the ICHM.

### Study variables

To calculate mortality and other rates and to describe age and gender proportions for the population aged 60+ from 2005 through 2012, publicly available data was collected from Statistics Norway (SSB) [35]. Patient characteristics are predictors of use of health care services. Therefore, age and gender were included as adjustment variables, as were diagnosis-related group (DRG) weight during the index stay (in the cohort) and functional status (in the subgroup). Additionally, the analyses were adjusted for year in consideration of time effect. The DRG weight indicates the amount of hospital resources required to treat the patients within a DRG category, and was used as a proxy for the complexity of disease. Functional status represents each person’s care needs and was calculated as a weighted mean value of 17 activities of daily living (ADL) variables that were measured during the follow-up year. The ADL variables were routinely recorded by the primary health care staff using a five-point scale, where 1 indicated self-reliant and 5 indicated that the patient was in need of extensive assistance. In some cases, a score of 9 was used to indicate that the variable was not applicable [36].

To calculate the weighted mean for each of the 17 ADL variables, all of (the daily) scores for the year following the index hospital discharge date were included for each participant. If two or more different scores were recorded within the one year-period (i.e., the score changed), each score was weighted by calculating the number of days the score was recorded. Thus, each score was multiplied by a number of days, and the products were added together, and then divided by the total number of days with any recorded score. When a value of 9 was given, the score was replaced by the previous value carried forward. When the previous value was also missing (or 9), then the next following value was used. In a small number of cases, a variable was assigned a value of 9 or missing for the whole period. Such particular ADL scores were treated as missing values. Finally, the mean value for the ADL variables was calculated for each person.

The total hospital use was calculated within each calendar year. The outcome variables for the cohort and the subgroup were calculated from the patients’ first stay (i.e., the index stay) for each of the years from 2008 through 2011. The discharge date was used to define the follow-up year, which began at index discharge and ran for one year.

A readmission is defined as any acute admission to the local general hospital within 30 days from the previous discharge [37,38]. For readmissions within 30 days during one year, the first subsequent readmission was counted, and could then become a new admission (i.e., date of discharge) from which a further readmission might occur [39,40].

The variable hour-based primary health care services includes home care nursing, practical assistance at home, day center visits, and other types of support to persons living at home.

### Analysis

Due to lack of registration in the municipalities, the ADL scores were missing for 36 patients in the subgroup who had received primary health care services (1.3% the ICHM and 1.5% in CM). The missing values were replaced by the weighted mean functional status of the subgroup receiving primary health care each year (ICHM: 2.2 in 2008, 2010 and 2011 and 2.3 in 2009. CM: 2.0 in 2008 and 2.3 in 2009–2011).

The ADL variables were not measured for 10.6% (157 patients) from the ICHM because these patients received only intermediate care and no other primary health care services. The missing values for this group were replaced by the average of the ADL scores for 36 individuals who were part of another study that investigated a population that was included in and partially overlapped with the subgroup in this study. The 36 individuals were also discharged from the local general hospital to intermediate care and did not receive other primary health care services during one year follow-up. Their ADLs were measured at discharge from the ICH and 3 and 6 months after index hospital discharge. The average functional status for the three points of time was calculated to 1.36. Replacing the missing values (for patients with only ICH) with 1.36 resulted in a decrease of the weighted mean functional status of 0.1 (i.e. increased functionality) for the ICHM-patients in the subgroup in 2009–2011. As a sensitivity analysis, functional status scores of 1 and 2 were also tested. These values caused only small changes (data not shown); i.e. the overall difference in use of primary health care services remained the same.

The analyses were age and gender standardized using the direct method [41]. The population of Nord-Trøndelag County, which has approximately 134,000 inhabitants, was chosen as the reference population. This county is divided into 23 municipalities, including the ICHM and the CM.

Descriptive statistics were used to describe the samples. For categorical variables, Pearson’s chi-square tests were used to identify any differences in proportions between the two municipalities. The Student’s t-test was used for continuous variables. Between-group differences were analyzed using analysis of covariance (ANCOVA). A pooled analysis of annual utilization for 2008–2011, with age, gender, year (categorized 1–4), in addition to DRG weight (in the cohort) and functional status (in the subgroup) as covariates, was conducted.

Only minor differences between crude values and adjusted estimates for all outcome variables regarding the use of general hospital were observed. For primary health care use, the adjustment increased the estimated outcomes in the ICHM while the opposite occurred in the CM. Furthermore, an ANCOVA for each of the follow-up years was also carried out (results mainly reported in figures). A significance level of 5% (p < 0.05) was chosen. The analysis was performed using SPSS 21.0 for Windows (IBM Corp., Armonk, NY).

## Results

### Total hospital use (Aim 1)

**Figure 3:**
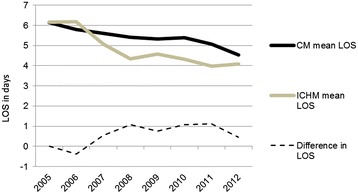
**Total hospital length of stay.** Mean and difference* in age-standardized length of stay (LOS), in days, in Norwegian hospitals from 2005–2012, for inhabitants aged 60+ residing the ICHM and the CM. Source: NPR. ICHM (Intermediate Care Hospital Municipality). CM (Comparative Municipality). *Positive values for this difference represent a lower LOS in the ICHM.

Figure 3 shows that during the two years prior to the introduction of the ICH (i.e., 2005–2006), the mean length of hospital stay (LOS) for patients living in the ICHM tended to be longer than that for patients living in the CM (0 to 7% longer). In 2007, when the ICH was established, the ICHM had a 10% shorter LOS than the CM (mean LOS: ICHM = 5.1 days, CM = 5.6 days). In 2008, when the ICH was fully operational, this difference increased to 20% (mean LOS: ICHM = 4.3 days, CM = 5.4 days). During subsequent years (2009–2012), the LOS of the ICHM was between 10% and 22% lower than that of the CM. Hence, the LOS was rapidly reduced after the introduction of intermediate care in the ICHM and remained lower than the LOS in the CM.

### Characteristics of the cohort at index stay

Between 17% and 19% of the inhabitants aged 60+ from the CM were admitted to the medical or surgical department of the local general hospital each year from 2008 to 2011, compared to 15%-16% of the inhabitants from the ICHM (Figure 2).

The mean age in the cohort in both municipalities was 76 years for the entire study period (Table 1). The proportion of females was lower in the CM (48.6%) than in the ICHM (54.0%, p = 0.054) in 2011; for the other years, the gender distribution was similar. The mean DRG weight during the index stay was also similar.

**Table 1:** **The cohort**

		**2008**	**2009**	**2010**	**2011**
N	CM	562	571	524	625
	ICHM	677	673	680	695
Female (n (%))	CM	300 (53.4%)	273 (47.8%)	252 (48.1%)	304 (48.6%)
	ICHM	350 (51.7%)	348 (51.7%)	347 (51.0%)	375 (54.0%)
Age (mean (SD) (median, range))	CM	76.1 (9.6) (77, 60 to 101)	75.7 (10.0) (76, 60 to 101)	75.9 (9.9) (75, 60 to 98)	76.0 (9.8) (75, 60 to 100)
	ICHM	75.8 (9.5) (76, 60 to 100)	75.6 (9.4) (76, 60 to 99)	75.6 (9.5) (75, 60 to 99)	76.1 (9.4) (76, 60 to 100)
No of diagnosis (mean (SD) (median, range))	CM	3.6 (1.9) (3, 1 to 8)	3.7 (2.0) (3, 1 to 8)	3.8 (2.0) (4, 1 to 10)	4.0 (2.2) (4, 1 to 16)
	ICHM	3.6 (1.8) (3, 1 to 8)	3.7 (1.9) (3, 1 to 8)	3.8 (2.2) (3, 1 to 16)	4.0 (2.3) (4, 1 to 15)
DRG-weight (mean (SD) (median, range))	CM	1.27 (1.08) (1.00, 0.12 to 6.70)	1.38 (1.99) (0.95, 0.15 to 23.94)	1.40 (1.53) (1.03, 0.03 to 23.58)	1.35 (1.13) (0.98, 0.03 to 7.36)
	ICHM	1.18 (0.98) (0.94, 0.18 to 4.47)	1.36 (1.93) (0.85, 0.15 to 23.94)	1.34 (1.64) (1.03, 0.02 to 23.58)	1.31 (1.32) (0.96, 0.03 to 22.40)
Acute admissions (n (%))	CM	473 (84.2%)	492 (86.2%)	445 (84.9%)	518 (82.9%)
	ICHM	582 (86.0%)	562 (83.5%)	584 (85.9%)	581 (83.6%)
Medical DRG (n (%))	CM	437 (77.8%)	445 (77.9%)	391 (74.6%)	469 (75.0%)
	ICHM	523 (77.3%)	492 (73.1%)	534 (78.5%)	536 (77.1%)

Characteristics of the cohort of hospitalized patients aged 60+ at index hospital stay (local general hospital) from the ICHM and the CM, 2008–2011.

ICHM (Intermediate Care Hospital Municipality). CM (Comparative Municipality).

A total of 83% to 86% of the admissions were acute, and the mean number of diagnoses assigned was approximately 4 in both municipalities. Between 73% and 78% of the patients were classified with a medical DRG, and the remaining patients were classified with a surgical DRG. The six most frequent major diagnostic categories (MDCs) in both municipalities were: musculoskeletal system and connective tissue, circulatory system, respiratory system, digestive system, nervous system, and kidney and urinary tract. Hence, only minor variations in patient characteristics were observed between the municipalities at the index stay.

### Characteristics of the subgroup

Between 8% and 10% of the inhabitants who resided in the CM and 8%-9% in the ICHM received primary health care services during the one year follow-up after their index hospital stay (2008–2011) (Figure 2). The mean age for the subgroup in both municipalities was approximately 80 years, and between 55% and 62% of the patients were females (Table 2). A difference of the yearly mean functional status score was observed in 2010 and 2011, when patients from the ICHM had a better functional status (2.1) than patients from the CM (2.3, p = 0.044). Otherwise, only minor variations in personal characteristics were observed between the municipalities in the subgroup.

**Table 2:** **The subgroup**

		**2008**	**2009**	**2010**	**2011**
N	CM	304	295	269	297
	ICHM	366	366	375	376
Female (n (%))	CM	187 (61.5%)	166 (56.3%)	150 (55.8%)	171 (57.6%)
	ICHM	222 (60.7%)	213 (58.2%)	207 (55.2%)	223 (59.3%)
Age (mean (SD) (median, range))	CM	80.8 (8.5) (83, 60 to 101)	80.6 (9.3) (83, 60 to 101)	81.3 (9.2) (83, 60 to 98)	81.3 (9.5) (83, 60 to 100)
	ICHM	80.5 (8.4) (82, 60 to 100)	79.5 (8.9) (81, 60 to 99)	80.3 (8.8) (82, 60 to 99)	79.9 (9.3) (81, 60 to 100)
Functional status (mean (SD) (median, range))	CM	2.0 (0.8) (2, 1 to 4)	2.3 (0.9) (2, 1 to 5)	2.3 (0.9) (2, 1 to 5)	2.3 (0.9) (2, 1 to 5)
	ICHM	2.2 (0.9) (2, 1 to 5)	2.2 (0.9) (2, 1 to 5)	2.1 (0.9) (2, 1 to 5)	2.1 (0.9) (2, 1 to 5)

Characteristics of the subgroup of persons aged 60 + in the ICHM and the CM receiving primary health care after index discharge from the local general hospital, 2008–2011.

ICHM (Intermediate Care Hospital Municipality). CM (Comparative Municipality).

Between 52% and 63% of the patients in the ICHM subgroup were admitted to the ICH each year. The age and gender of these patients were similar to those of the entire ICHM subgroup (mean age 79, 59% female), but the mean functional status of these patients was slightly better than that of the entire subgroup (2.0-2.1 in the ICH).

### Readmissions to the local general hospital (Aim 2)

For the follow-up years 2008–2011, only minor and non-significant differences were observed between the municipalities for the outcomes “proportion of patients with readmission within 30 days after the index discharge”, “Mean number of readmission incidents within 30 days after index discharge” and “Mean number of readmission incidents (within 30 days) during one year” (Table 3).

**Table 3:** **Local general hospital utilization**

**Variable**	**Municipality**	**Crude mean (95% CI)**	**Est. mean (95% CI)**
Proportion of patients with readmission within 30 days after index discharge	CM	0.14 (0.13 to 0.16)	0.14 (0.13 to 0.16)
	ICHM	0.13 (0.12 to 0.14)	0.13 (0.12 to 0.14)
Mean number of readmission incidents within 30 days after index discharge	CM	0.16 (0.14 to 0.18)	0.16 (0.14 to 0.18)
	ICHM	0.16 (0.14 to 0.17)	0.16 (0.14 to 0.17)
Mean number of readmission incidents (within 30 days) during 1 year	CM	0.34 (0.31 to 0.38)	0.34 (0.31 to 0.38)
	ICHM	0.34 (0.30 to 0.37)	0.34 (0.30 to 0.37)
Mean number of admissions 1 year	CM	1.91 (1.85 to 1.97)	1.91 (1.85 to 1.97)
	ICHM	1.89 (1.83 to 1.94)	1.89 (1.84 to 1.95)
- Acute admissions	CM	1.64 (1.58 to 1.69)	1.64 (1.58 to 1.69)
	ICHM	1.60 (1.55 to 1.66)	1.61 (1.55 to 1.66)
Mean length of index stay (days)	CM	5.2 (5.0 to 5.5)	5.2 (5.0 to 5.4)
	ICHM	3.9 (3.6 to 4.1)	3.9 (3.7 to 4.1)
Mean number of hospital days 1 year	CM	10.6 (10.1 to 11.1)	10.5 (10.1 to 11.0)
	ICHM	7.5 (7.1 to 8.0)	7.6 (7.2 to 8.0)

Use of local general hospital one year from index stay 2008–2011 for the cohort of patients aged 60+ from the ICHM and the CM.

ICHM (Intermediate Care Hospital Municipality). CM (Comparative Municipality).

Analysis with ANCOVA adjusted for age, gender, DRG weight at index hospital stay and year (categorized 1–4).

### Use of the local general hospital (Aim 3)

With respect to the assessment of hospital stays, only minor differences in the number of admissions or acute admissions were observed between the two municipalities in the follow-up years 2008–2011 (Table 3). The mean length of the index stay was statistically significantly lower for patients who resided in the ICHM (estimated mean difference = 1.3 days per hospitalized patient and year, p < 0.001). Patients from the ICHM also spent fewer days in the hospital during one year than patients from the CM (estimated mean difference = 2.9 days per hospitalized patient and year, p < 0.001).

**Figure 4:**
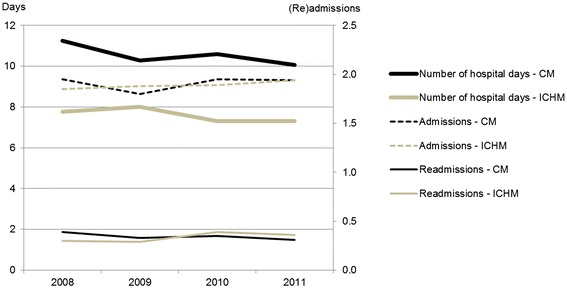
**Local general hospital utilization each year.** Mean number of number of hospital days, admissions and readmissions to the general hospital during one year after index hospital stay for the cohort of patients aged 60+ from the ICHM and the CM. The index year (2008–2011) defines the follow-up year. ICHM (Intermediate Care Hospital Municipality). CM (Comparative Municipality). Analysis with ANCOVA adjusted for age, gender and DRG weight at index hospital stay.

An analysis for each year revealed similar results as the pooled analysis presented above. This is illustrated in Figure 4.

### Use of primary health care services after hospital discharge (Aim 3)

Patients in the ICHM received a statistically significant larger number of hour-based primary health care services after hospital discharge compared to patients in the CM in the follow-up period 2008–2011 (estimated mean difference = 59 hours per patient and year, p < 0.001) (Table 4).

**Table 4:** **Primary health care utilization after hospitalization**

**Variable**	**Municipality**	**Crude mean (95% CI)**	**Est. mean (95% CI)**
Mean number of hour-based primary health care services	CM	181 (159 to 204)	175 (154 to 196)
	ICHM	229 (209 to 249)	234 (215 to 252)
Mean number of days in institutional care	CM	51.8 (46.4 to 57.2)	49.7 (44.9 to 54.6)
	ICHM	35.4 (30.6 to 40.2)	37.0 (32.7 to 41.3)
- Short-term institutional care	CM	16.9 (15.0 to 18.7)	16.5 (14.7 to 18.3)
	ICHM	14.9 (13.3 to 16.5)	15.2 (13.6 to 16.7)
- Nursing home (long-term)	CM	35.1 (30.3 to 39.8)	33.3 (29.0 to 37.7)
	ICHM	20.5 (16.3 to 24.8)	21.9 (18.0 to 25.7)
Mean number of days in adapted residence	CM	45.6 (39.2 to 52.0)	43.8 (37.6 to 50.0)
	ICHM	42.3 (36.6 to 48.0)	43.7 (38.2 to 49.1)

Use of primary health care one year after index hospital stay 2008–2011 for the subgroup of persons aged 60+ in the ICHM and the CM.

ICHM (Intermediate Care Hospital Municipality). CM (Comparative Municipality).

Analysis with ANCOVA adjusted for age, gender, functional status (weighted mean during the follow up year) and year (categorized 1–4).

The CM offered in average 12.7 more days in institutional care in the year after hospital discharge than the ICHM (days in ICH included) (p < 0.001). Divided into short- and long-term stays, the estimated mean difference was 11.4 more days of long-term stays in nursing homes per patient and year in the CM (p < 0.001). No significant difference in days spent in short-term institutional care or adapted residence was observed between the municipalities.

**Figure 5:**
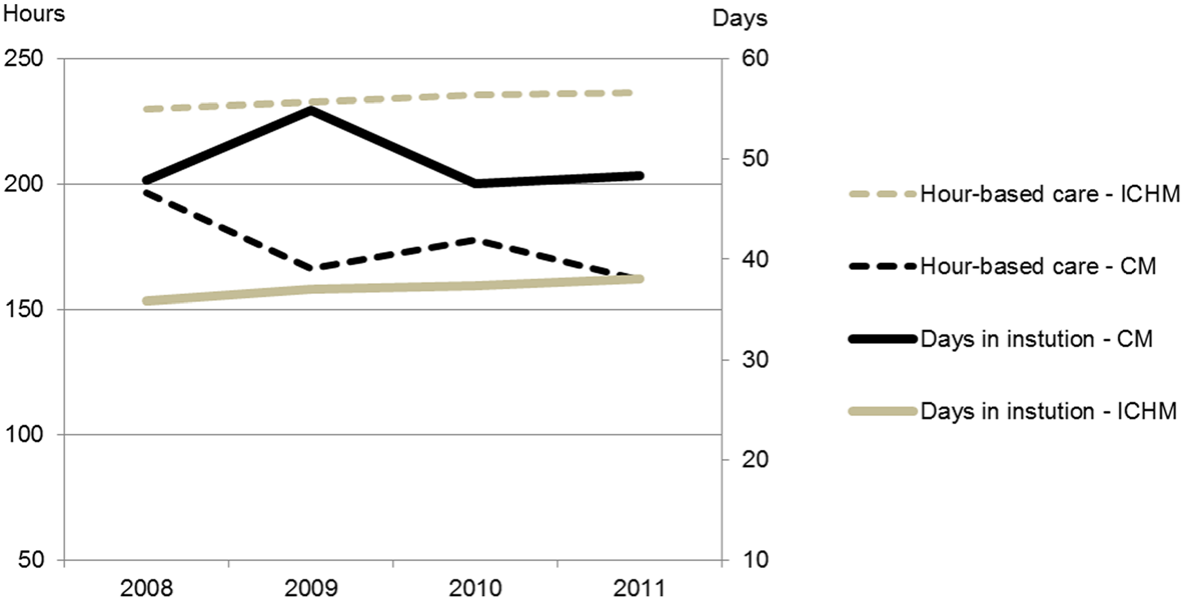
**Primary health care utilization each year.** Mean number of hour-based care and days in institutional care one year after index hospital stay for the subgroup of persons aged 60+ in the ICHM and the CM. The index year (2008–2011) defines the follow-up year. ICHM (Intermediate Care Hospital Municipality). CM (Comparative Municipality). Analysis with ANCOVA adjusted for age, gender and functional status (weighted mean during the follow up year).

An analysis for each year was also conducted for the subgroup and the results did not substantially deviate from the pooled analysis (Figure 5).

**Figure 6:**
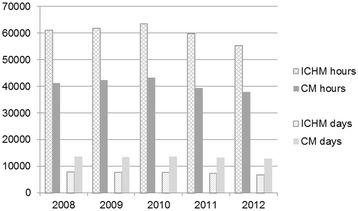
**Primary health care utilization for all inhabitants aged 60+.** Age-standardized number of days in institutional and hour-based primary health care per 1,000 inhabitants aged 60+ residing in the ICHM and the CM in 2008–2012. ICHM (Intermediate Care Hospital Municipality). CM (Comparative Municipality). Hour-based primary health care services include home care nursing, practical assistance at home, day center visits, and other types of support to persons living at home. Institutional days are short- and long-term stays in primary health care included the ICH (Intermediate Care Hospital) in the ICHM.

Figure 6 shows a steady utilization of days and hour-based primary health care services for all inhabitants aged 60+ in both municipalities in the observed period. A comparison of the total hours and institutional days offered reveals that inhabitants aged 60+ in the ICHM received 48% more hour-based care than these inhabitants in the CM from 2008 to 2012. In contrast, the use of institutional days (short- and long-term) was 43% lower in the ICHM than in the CM (Figure 6).

### Mortality

**Figure 7:**
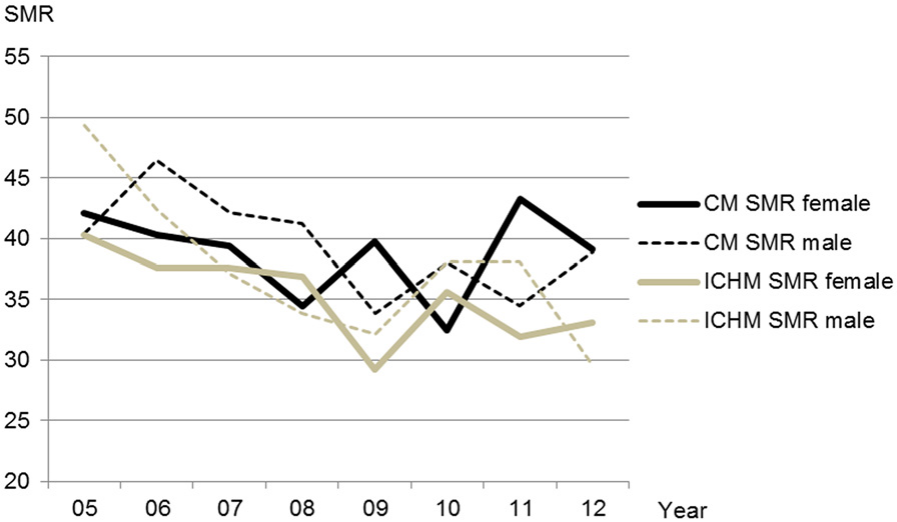
**Mortality for all inhabitants aged 60+.** Standardized mortality rate (SMR) for men and women aged 60+ in the ICHM and in the CM 2005–2012. ICHM (Intermediate Care Hospital Municipality). CM (Comparative Municipality).

Only minor variations in the mortality rates for men and women were observed between the municipalities from 2005 to 2012 (Figure 7). A comparison revealed that the total yearly mortality rates for inhabitants aged 60+ were slightly lower in the municipality with the ICH, with the exception of 2005 and 2010.

## Discussion

From 2005 to 2012, the total hospital admission rates in the two municipalities were similar. The length of hospital stay (LOS) decreased after the ICH was introduced (2007) and remained between 10% and 22% lower in the ICHM than in the CM, without an increase in readmissions. Only minor changes in the municipalities’ utilization of primary health care services occurred in the years after the establishment of the ICH.

### Strengths and limitations

The strengths of this study were the inclusion of complete and comparable large populations that were observed for several years, the adjustment of the analyses for differences in the characteristics of the groups and the collection of data from reliable electronic registers.

The main limitation of this study is the design, which compares two municipalities that have organized their health care services somewhat differently; this practice is common in Norway [31,32]. The comparative municipality was selected purposefully based on the presence of many similar characteristics, and the analyses were adjusted to make the comparison more reliable. Nevertheless, the effect of other factors cannot be completely ruled out. The generalizability of the findings to other municipalities in Norway depends on the local circumstances. However, we believe that the results of this study can be generalized to similar municipalities in Norway and to other similar settings.

Another potential limitation of this study is that only readmissions to the same local general hospital were included. However, readmissions for patients (≥18 years) in Norway are primarily to the same hospital as the index admission; for the local general hospital, this rate was 93% in 2008 and 2009 [42]. This finding indicates that the study design accounts for most of the readmissions.

### Hospital utilization

The LOS has decreased over the past decade in all European countries [43], and this trend is also apparent among elderly patients in Norway [44]. The most apparent finding in this study was that the LOS was reduced after the introduction of the ICH. A reduction in the LOS was also observed in the CM, but this reduction was more gradual than the reduction observed in the ICHM. In the ICHM, the LOS dipped below 5 days as early as 2008; in contrast, this threshold was first achieved in 2012 in the CM. Thus, the ICH was a rapid means of achieving reduced LOS.

The marked drop in LOS that was observed in the CM in 2011–2012 likely occurred due to the implementation of the Norwegian Coordination reform on January 1, 2012; this reform aimed to achieve earlier discharges using economic incentives [2]. However, it is also possible that the practice of earlier hospital discharges that was established in the ICHM spread to neighboring municipalities, leading to a culture change in the CM.

Readmission may reflect the total chain of care [38,45] and is increasingly being used as an indicator of quality of care [10]. In this study, the level of readmissions in the municipality with the ICH was similar to internationally [9,46] and nationally reported levels [42,47]. However, a short average LOS may increase the risk of early readmissions [48]. According to a Cochrane review [26], elderly people living in a municipality with an ICH could be at risk of increased readmissions. In the current study, no differences in readmissions were observed between the municipalities, alleviating the concern that a shorter LOS caused by transfer to an ICH increases readmissions [12]. This conclusion is further supported by the absence of a difference in the total number of acute admissions.

Finally, a shorter LOS could also raise concerns about greater mortality rates caused by premature hospital discharges. However, increased mortality rates were not observed in the ICHM in this study.

### Primary health care utilization

The utilization of short-term institutional stays after hospitalization was similar in the municipalities when including ICH stays in the ICHM, whereas the differences between the municipalities in long-term stays and hour-based care were significant. As this might be due to differences in the inhabitants’ functional status (worse functional status indicates need for institutional care), we did a supplementary analysis comparing functional status adjusted for age and gender each year. Functional status was the same except for 2011 where it was worse (i.e. higher value) in the CM (2.27 vs 2.11, p = 0.020). Hence, it seems reasonable to explain the observed differences in use of hour-based and institutional stays in primary health care by different local health politics and not by minor differences in functional status. Using hour-based services to enable elderly people to stay in their own homes as long as possible is in accordance with the current health policy in Norway [2]. Furthermore, the difference in use of institutional care may be dependent on the available institutional care beds which are considerable higher in the CM (1.5% for inhabitants in the ICHM aged 60+ compared to 3.4% in the CM).

The utilization of primary health care services in both municipalities remained fairly constant throughout the study period. Due to this stable pattern of utilization, it is likely that the ICH had a minor impact on the use of primary health care services during the follow-up years.

## Conclusions

During the years after the introduction of the ICH, the length of hospital stay for patients aged 60+ was reduced by between 10% and 22% without increasing the number of admissions or readmissions. This study provides no indications that early hospital discharge to a municipality offering follow-up in an ICH exposes patients to an increased health risk or to inadequate rehabilitation, neither during the discharge period nor in the long term.

Only minor changes in the two municipalities’ health care services occurred during the follow-up years. Hence, the ICH operated as an extension of the general hospital by acting as a substitute for some days that would otherwise be spent in the hospital; however, the ICH had a minor impact on the pattern of primary health care utilization.

## Abbreviations

ICHM: Intermediate Care Hospital Municipality (Stjørdal Municipality)
CM: Comparative Municipality (Verdal municipality)
ICH: Intermediate Care Hospital
LOS: Length of hospital stay
ADL: Activities of daily living
